# Trilayer Temporalis Fascia Interposition Graft for Nasal Septal Perforation Repair: A Continued Experience

**DOI:** 10.1002/ohn.1183

**Published:** 2025-03-10

**Authors:** Derek J. Vos, Kristen A. Echanique, Amit Nag, Stephen Hadford, Peter Ciolek, Dane J. Genther

**Affiliations:** ^1^ Cleveland Clinic Head and Neck Institute Cleveland Ohio USA; ^2^ Case Western Reserve University Cleveland Ohio USA; ^3^ Cleveland Clinic, Section of Facial Plastic and Microvascular Surgery Cleveland Ohio USA

**Keywords:** nasal septal perforation, perifascial areolar tissue, polydioxanone plate, temporalis fascia

## Abstract

**Objective:**

The repair of nasal septal perforation (NSP) is complex, with a variety of described techniques and reported outcomes. At our institution, we commonly perform NSP repair using a trilayer graft of thin polydioxanone (PDS) plate wrapped on both sides with temporalis fascia without intranasal flaps. We aim to report our continued experience with this technique.

**Study Design:**

Retrospective chart review.

**Setting:**

Single institution.

**Methods:**

Retrospective chart review of patients undergoing repair of NSP using a trilayer temporalis fascial interposition grafting technique at our institution from 9/1/2018 to 10/31/2023.

**Results:**

Fifty‐six patients (mean age 45 years, 58.9% female) were included in this study. The suspected cause of NSP was primarily iatrogenic (n = 26, 45.6%); however, a large number of patients did not have a definitive etiology of NSP (n = 18, 32.1%). The most commonly reported symptoms pre‐operatively included nasal obstruction/congestion (n = 51, 91.1%) and nasal crusting (n = 31, 55.4%). NSPs were most frequently anterior in location (n = 46, 82.1%) and medium in size (1‐2 cm) (n = 31, 55.4%), followed by small (<1 cm) (n = 17, 30.4%). All patients within this study experienced improvement in their pre‐existing symptoms associated with NSP, with complete resolution of prior symptoms occurring in the majority of patients (n = 44, 78.6%). A minority of patients in this cohort experienced postoperative complications (n = 8, 14.3%). One patient (1.8%) demonstrated persistent perforation following repair. The median length of follow‐up in this study was 257 days (range 65‐1724).

**Conclusion:**

The trilayer temporalis fascial interposition graft is an effective and reliable tool for the repair of NSP.

Nasal septal perforation (NSP) is a full‐thickness defects of the nasal septum caused by loss of mucoperichondrial lining and underlying cartilage or bone. They may arise from several possible etiologies, including iatrogenic injury from nasal surgery or cauterization, intranasal drug use, rheumatologic or inflammatory conditions, and trauma. Prevalence of this condition is estimated at <1%–3%, depending on the specific population studied.^1^, ^2^, ^3^ The clinical presentation of NSP is variable, ranging from asymptomatic to those causing significant derangements in nasal breathing and associated nasal symptoms of crusting, whistling, and epistaxis. As a result of these symptoms, patients with NSPs often exhibit a reduction in overall quality of life.^4^, ^5^

The management of NSPs varies and is dependent upon size, location, orientation, and symptomatology of the perforation. In patients without significant symptom burden, medical management alone consisting of a robust nasal hygiene regimen with daily intranasal saline sprays, topical emollients, and humidification of their local environment may be trialed. For patients with smaller perforations who are refractory to medical management and poor surgical candidates, nasal obturation in the form of a nasal septal button can be considered. However, for patients who desire a more permanent solution, surgical repair is often necessary.

Surgical repair of NSPs is complex and may be accomplished through a multitude of techniques with varying degrees of success. These techniques include both open and closed approaches and may range in complexity from local tissue rearrangement to free‐tissue transfer.^6^ At our institution, we routinely perform NSP repair using a trilayer graft, composed of a thin polydioxanone (PDS) plate wrapped with the superficial layer of the deep temporal fascia (temporalis fascia). We have previously reported on this technique and our initial experience with it, demonstrating a high degree of success.^7^ Given this success, we have continued to employ the trilayer temporalis fascial interposition graft for repair of NSPs and, thus, aim to report our continued experience with this technique, including lessons learned and additional insights.

## Methods

### Data Collection and Analysis

This study was approved by the Institutional Review Board of the Cleveland Clinic Foundation. A retrospective chart review of all patients undergoing NSP repair using the trilayer temporalis fascial interposition grafting technique at our institution from 9/1/2018 to 10/31/2023 was conducted. Patients who underwent NSP repair using an alternative technique were excluded, as well as those patients with less than 60 days of postoperative follow‐up. Patient demographics were collected and included age, sex, race, current or past nicotine use, and intranasal drug use. Additional variables collected included etiology of the perforation, preoperative symptomatology, duration of perforation, prior nasal surgery, or intranasal steroid use.

NSPs were characterized by size in a similar fashion to that reported by Delaney et al^8^: small (<1 cm), medium (1‐2 cm), and large (>2 cm).

Qualitative variables were compared using Chi‐Fisher's exact test. Statistical analysis was performed on R studio with *P* < 0.05 used to indicate a statistically significant difference.

### Surgical Technique

All patients underwent septal perforation repair using the technique as originally described by Hadford et al.^7^ In brief, an open septorhinoplasty approach is used to gain access to the septum. Often, additional functional septorhinoplasty procedures are concurrently performed when necessary. A transcolumellar open approach is used to elevation the skin‐soft tissue envelope, and the septum is approach by release of the upper lateral cartilages and elevation of bilateral mucoperichondrial flaps in the standard fashion to the level of the perforation. Once reached, the perforation is opened sharply and elevated circumferentially. Any residual cartilage or bone along the perimeter of the perforation is removed. Next, a temporalis fascia graft of up to 6 cm is harvested through a 3 to 4 cm incision in the hairline between the branches of the superficial temporal vessels. The plane is carried down to the superficial layer of the deep temporal fascia. The fascia is then incised and freed of the underlying temporalis muscle and set aside. Liposomal bupivacaine‐soaked gel foam is then placed in the harvest site for prolonged local anesthesia.

Next, the trilayer temporalis fascia PDS plate graft is constructed using a perforated 0.15 mm PDS plate. The plate is cut to size several mm larger than the defect and wrapped in the harvested temporalis fascia to create a trilayer graft. The fascia is secured to the PDS plate using several 5‐0 PDS sutures along the periphery. Constructing the graft to be larger than the perforation allows for adequate overlap of the graft with healthy mucosa and increases the rate of mucosalization and incorporation of the graft.

**Figure 1:**
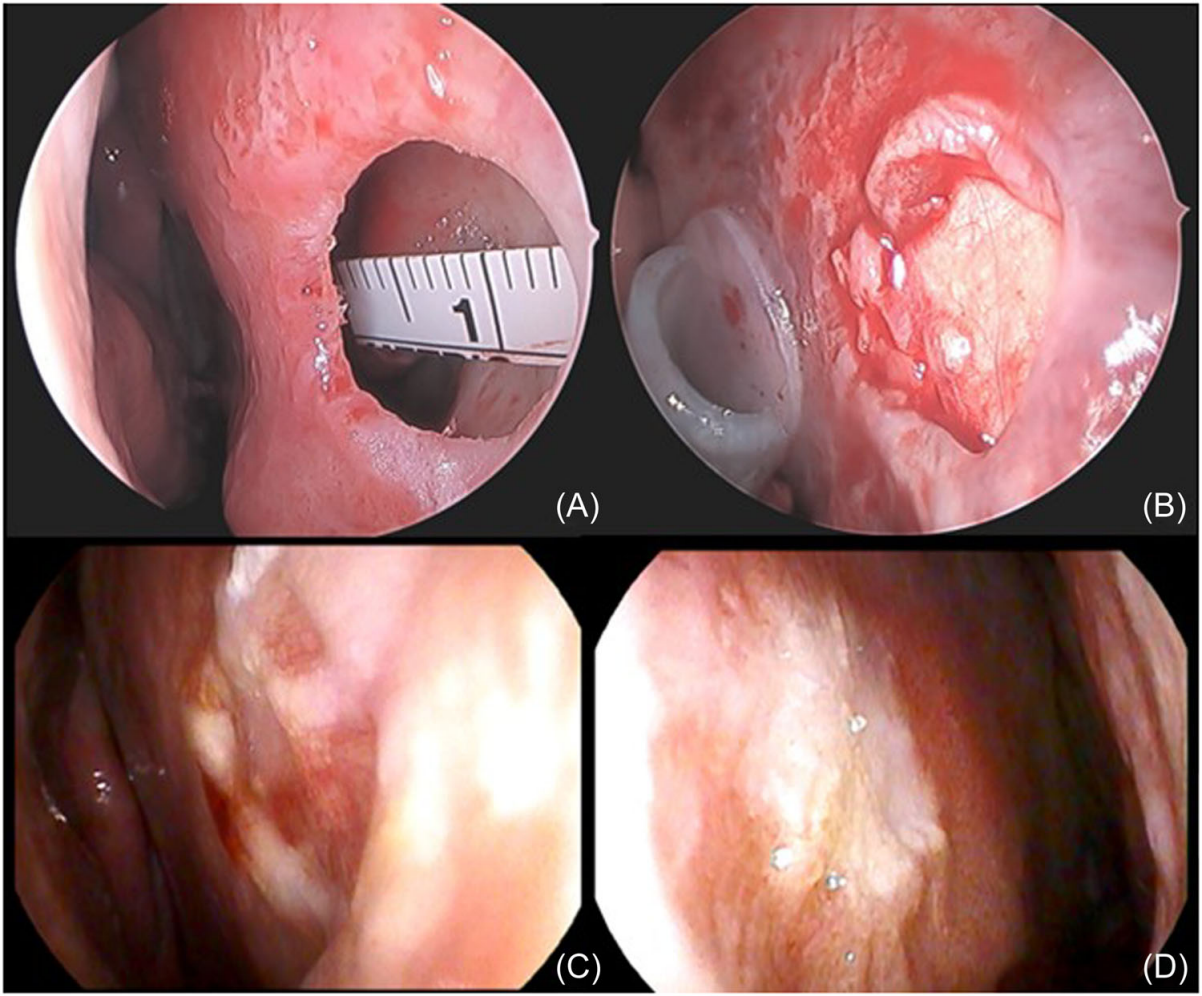
(A) Intra‐operative photograph demonstrating a medium‐sized anterior septal perforation. (B) Intra‐operative photograph demonstrating graft in place. (C) and (D) Post‐operative photographs taken at 2 weeks post‐operatively, demonstrating revascularization and healing of the graft.

The graft is then placed between the mucoperichondrial flaps as an interposition graft and secured to the native strut using a single 5‐0 PDS suture. The mucosal edges are redraped to ensure that the circumference of the perforation is reconstructed with the trilayer graft. If the perforation is located near the floor of the nose, elevation of the mucosa is extended along the floor laterally to allow for adequate overlap with the inferior portion of the graft. Importantly, no local flaps are utilized in this technique. Intra‐operative photos demonstrating NSP pre‐ and post‐repair are shown in Figure 1. Modified Doyle splints without the tubed component are then carefully placed and secured to the membranous septum with a Prolene suture. Splints remain in place for two weeks, and patients are discharged home on a postoperative course of antibiotics.

## Results

During the study period specified, a total of 56 patients (mean age 45 years, 58.9% female) underwent NSP repair using trilayer temporalis fascial interposition grafting technique and met inclusion criteria (Table 1). The suspected cause of NSP was primarily iatrogenic (n = 26, 45.6%); however, a large number of patients did not have a definitive etiology of NSP (n = 18, 32.1%). Each patient in this study was symptomatic prior to surgical repair, with the most commonly reported symptoms including nasal obstruction/congestion (n = 51, 91.1%), nasal crusting (n = 31, 55.4%), whistling (n = 21, 37.5%), and frequent epistaxis (n = 18, 32.1%). The mean duration of NSP was 33.2 months. The majority of patients had a history of prior nasal surgery (n = 32, 57.1%) or history of intranasal steroid use prior to surgery (n = 35, 62.5%).

**Table 1:** Demographic Details

Age at repair, mean ± SD	45 ± 16.8
Sex, n (%)	
Male	23 (41.1)
Female	33 (58.9)
Race, n (%)	
White	53 (94.6)
Asian	2 (3.6)
Black	1 (1.8)
Suspected cause of perforation, n (%)	
Iatrogenic	26 (46.4)
Unclear	18 (32.1)
Other	12 (21.4)
Pre‐operative symptoms, n (%)	
Nasal obstruction	51 (91.1)
Crusting	31 (55.4)
Whistling	21 (37.5)
Epistaxis	18 (32.1)
Duration of perforation, months, mean ± SD	33.2 ± 56.6
Previous nasal surgery, n (%)	32 (57.1)
Previous intranasal steroids, n (%)	35 (62.5)
Previous intranasal oxymetazoline, n (%)	4 (7.1)
Previous nicotine use, n (%)	16 (28.6)
Accompanying nasal surgery, n (%)	46 (82.1)
Repair vestibular stenosis	39 (69.6)
Septoplasty	36 (64.3)
Inferior turbinate reduction	29 (51.8)
Size of perforation, n (%)	
Small (<1 cm)	17 (30.4)
Medium (1–2 cm)	31 (55.4)
Large (>2 cm)	8 (14.3)
Location of perforation, n (%)	
Anterior	46 (82.1)
Other	10 (17.9)
Duration of postoperative splinting, mean ± SD	14.7 ± 6.0
Duration of postoperative antibiotics, mean ± SD	7.9 ± 3.3
Length of follow‐up, mean ± SD	405 ± 426.2

At the time of surgery, NSP repair using trilayer temporalis fascia interposition grafting predominantly accompanied other nasal procedures, including repair of nasal vestibular stenosis (n = 39, 69.6%), septoplasty (n = 36, 64.3%), and inferior turbinate reduction (n = 33, 58.9%). NSPs were most frequently anterior in location (n = 46, 82.1%) and medium in size (1‐2 cm) (n = 31, 55.4%), followed by small (<1 cm) (n = 17, 30.4%). Following repair, Doyle splints were kept in place for a mean of 14.7 days. Postoperative antibiotics were continued for an average of 7.9 days.

All patients within this study experienced an improvement in their pre‐existing symptoms associated with NSP. Specifically, the majority of patients experienced complete resolution of their prior symptoms (n = 44, 78.6%), with the remaining 12 patients (21.4%) experiencing partial resolution. In patients who did not experience complete resolution of symptoms, nasal obstruction was the most cited persistent symptom (n = 9), followed by crusting (n = 3). When examining patients with persistent nasal obstruction, septal thickening was noted on nasal endoscopy in 77.8% (n = 7). Importantly, no patients with persistent nasal obstruction required additional surgical intervention to address these symptoms. Among patients with persistent crusting, one patient reported continued nasal picking, and another was noncompliant with the use of nasal irrigations/emollient post‐operatively.

One patient within the study demonstrated the persistence of a partial NSP following the trilayer temporalis fascia interposition graft. This was discovered at the patient's four‐month post‐operative appointment. Despite this, the patient experienced partial improvement in symptoms, reduction in size of perforation, and ultimately did not elect to pursue additional repair. When examining factors associated with lack of complete symptom resolution (Table 2), no statistically significant relationships between patient age, suspected cause of perforation, size of perforation, location of perforation, prior nasal surgery, prior nasal steroid use, concomitant nasal procedure, duration of antibiotics, or duration of splinting was found.

**Table 2:** Factors Associated With Partial Resolution of Symptoms and Postoperative Complications

	Partial resolution of symptoms, *n* (%)	*P*	Post‐operative complication, *n* (%)	*P*
Cause of perforation				
Iatrogenic	6 (10.7)	1	3 (5.4)	0.7116
Unclear	2 (3.6)	0.3001	2 (3.6)	1
Other	4 (7.1)	0.2626	3 (5.4)	0.3483
Prior nasal surgery	8 (14.3)	0.5254	3 (5.4)	0.268
Prior nasal steroids	9 (16.1)	0.5027	2 (3.6)	0.0423
Prior nicotine use	3 (5.4)	1	4 (7.1)	0.2063
Accompanying nasal surgery	11 (19.6)	0.6715	7 (12.5)	1
Anterior perforation	9 (16.1)	0.4326	8 (14.3)	0.3265
Size of perforation				
Small	2 (3.6)	0.3092	2 (3.6)	1
Medium	8 (14.3)	0.5163	5 (8.9)	0.7198
Large	2 (3.6)	1	1 (1.8)	1
Antibiotic duration >7 d	2 (3.6)	1	4 (7.1)	0.0553
Splints >7 d	11 (19.6)	0.672	5 (8.9)	0.0778
Age >45	9 (16.1)	0.1043	4 (7.1)	1

A minority of patients in this cohort experienced postoperative complications (n = 8, 14.3%). These included post‐operative infection (n = 3, 5.4%), synechiae requiring lysis (n = 2, 3.6%), nasal hemorrhage (n = 1, 1.8%), incomplete repair of the perforation (n = 1), delayed healing presenting as septal thickening and cartilage irregularity (n = 1, 1.8%), and dehiscence of the temporalis fascia harvest site (n = 1). Patients who did not have a history of prior intranasal steroid use (n = 21, 37.5%) had a statistically higher rate of complication (p = 0.0423) (Table 2). No patients required revision trilayer grafting for repair of NSP. The median length of follow‐up in this study was 257 days (range 65‐1724).

## Discussion

Patients with NSPs often experience significant symptoms that are associated with reduced quality of life and pose a challenge for both the patient and surgeon. Furthermore, many well‐established techniques are technically challenging and vary in their rates of success. At our institution, we routinely perform repair of NSP using a trilayer temporalis fascia interposition graft with PDS plate. The present study explores our continued experience with this technique over a 5‐year period. Importantly, this study represents a very consistent, standardized technique amongst all patients included within.

Many techniques exist for the repair of NSPs, including a range of permutations of mucosal flaps and pedicled flaps, interposition grafts, and vascularized free tissue transfer. Both temporalis fascia grafts and PDS plates have been independently described for the repair of NSP, with varying degrees of success. In a study of 75 patients with NSP by Miglani et al, the use of temporalis fascia alone as an interposition graft was found to successfully resolve perforations of an average of 1.2 × 1.7 cm in all patients; however, 12% of patients demonstrated persistent nasal obstruction which required revision surgery to address.^9^ In our experience, the use of temporalis fascia alone is cumbersome to work with due to its lack structural rigidity, necessitating numerous sutures to secure it in place between the septal flaps. In contrast, a study by Sand et al compared the use of bilateral mucoperichondrial flaps with various interposition grafts, including PDS plate with temporalis fascia, finding similar perforation repair outcomes for small perforations, with the combination of PDS and temporalis fascia grafting providing superior outcomes for medium and large perforations.^10^

An additional technique described in the literature for the repair of NSP is the use of a PDS plate construct in combination with a temporoparietal fascia (TPF) graft. While similar to our technique, the use of TPF over temporalis fascia results in a thicker graft, which may be associated with complications such as nasal obstruction. A study of 31 patients undergoing NSP repair using PDS plate with TPF grafting found an overall success rate of 90% with a 22% complication rate.^11^ These complications included three instances of persistent perforation, seven instances of nasal crusting, and one episode of infection. A multi‐institutional study of 17 patients using a similar technique found 100% resolution of perforation, with 47% of patients undergoing postoperative complications in the form of pain, nasal obstruction/crusting, and fascial harvest site complications.^12^ This technique has been further adapted and used in a closed fashion using a hemitransfixion incision, with 90% resolution of NSP.^13^ Our use of temporalis fascia instead of temporoparietal fascia may confer the benefit of a thinner flap and the presence of a perifascial vascular plexus, potentially leading to improved outcomes.

Complete resolution of symptoms associated with NSP was achieved in the majority of patients within our study. Interestingly, patients who experienced continuous nasal obstruction following trilayer temporalis fascia grafting were often noted on postoperative endoscopy to have edema or thickening of the nasal septum. In the literature, the use of PDS plates has been associated with an increased risk of septal thickening in a small subset of patients, likely secondary to increased inflammation.^14^, ^15^, ^16^ These studies specifically identify current nicotine use as a risk factor for complications associated with PDS plate repair of NSP. We suspect that the inflammation associated with the PDS plate causes a slight reduction in the size of the nasal airway and is likely contributing to patient's subjective experience of continued nasal obstruction. Importantly, the persistence of nasal obstruction, congestion, or crusting following NSP repair is not uncommon.^17^, ^18^, ^19^ In a recent study assessing the use of the nasal obstruction symptom evaluation (NOSE) score for NSP, it was found that 32.7% of patients reported moderate to severe nasal congestion post‐operatively, with 35.9% of patients reporting moderate to severe crusting at a mean follow‐up length of 9 months.^20^ When examining patients in our study who reported persistent crusting, lack of compliance with appropriate post‐operative nasal hygiene was noted in the majority of patients. This suggests the importance of optimizing nasal hygiene in the form of nasal irrigation, topical emollient, and/or humidification.

A primary advantage of this technique is that no mucosal flaps are required to achieve repair. Creating, mobilizing, and securing local flaps within the nose is often a time consuming and arduous process. The lack of intranasal flaps would, therefore, reduce operative time. While the use of temporalis fascia does confer the need for an additional surgical site, the site is accessible within the surgical field, and the incision is concealed within the hairline. Additionally, it confers low morbidity. Among all patients, only one mild harvest site incision dehiscence was noted in a patient who was an active nicotine user, and this was treated with conservative management. In a recent systematic review assessing the use of TPF in combination with PDS plating, two of the seven included studies reported complications at the harvest site, including temporal pain at 5‐6% and scalp seroma at 6%.^21^ Within our study, the rate of complication associated with temporalis fascia was very low, with only one patient exhibiting mild dehiscence of the surgical site. Our results, thus, indicate that the harvest of temporalis fascia is a well‐tolerated and safe procedure with very low instances of post‐operative complications.

**Figure 2:**
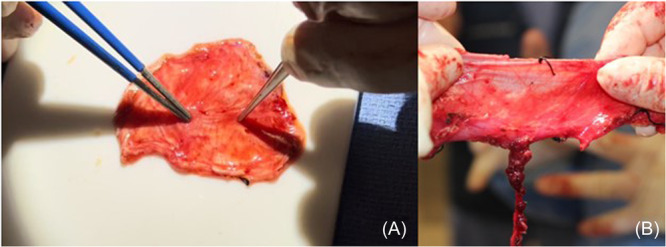
Perifascial areolar tissue (PAT). (A) PAT overlying the temporalis fascia harvested for the graft. (B) PAT overlying the fascia lata in an anterolateral thigh fascia lata free flap.

In addition to minimal donor site morbidity, our experience has led us to hypothesize that the success of our trilayer temporalis fascia interposition grafting may be attributed to the rich vascular plexus overlying the temporalis fascia that is incorporated into the graft, known as perifascial areolar tissue (PAT).^22^ This highly vascularized tissue is demonstrated in Figure 2A. In the literature, PAT has been demonstrated to have excellent survival even in ischemic environments, due to its abundance of mesenchymal stem cells.^22^, ^23^, ^24^ As a result of these tissue characteristics, the use of PAT as a grafting material has been well‐described within the literature, utilized frequently as a dermal or soft‐tissue substitute for coverage of defects of bone, tendon, or plates.^23^, ^25^, ^26^, ^27^ Our experience has shown that harvesting temporalis fascia along with its associated PAT can promote successful tissue healing over the avascular PDS plate and ultimately may lead to improved outcomes in the repair of NSPs. Future studies specifically examining the role of PAT and its rich vascular plexus associated with temporalis fascia grafting are needed to better quantify this relationship.

**Figure 3:**
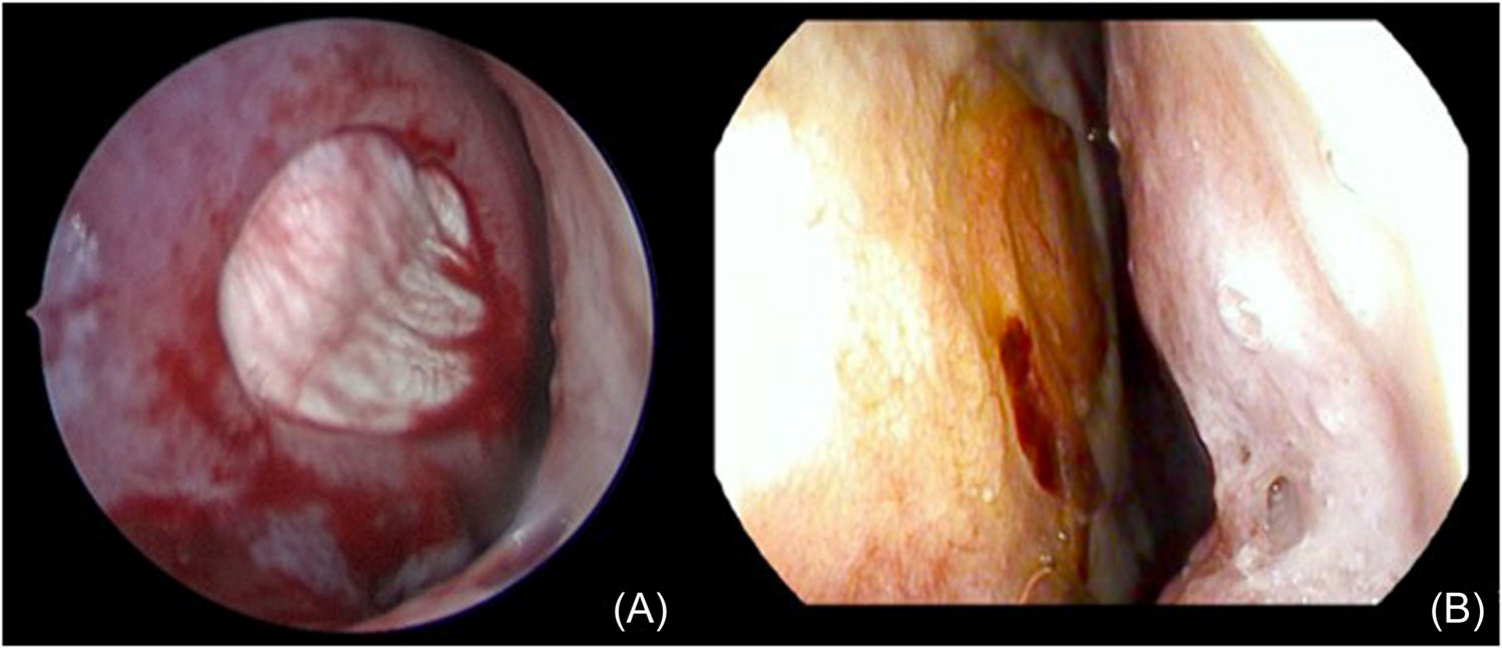
(A) Intra‐operative photo demonstrating fascial graft in place. (B) Endoscopic photo taken at two weeks post‐operatively, demonstrating successful recanalization of pre‐existing blood vessels within the graft and integration of fascial graft.

Given the demonstrated role of PAT in promoting neovascularization and successful tissue healing across a wide array of defects, it is not surprising that the use of temporalis fascia and its associated PAT provides an advantage over other NSP repair techniques. By harvesting fascial tissue with its overlying vascular plexus and associated PAT, we believe that patients may exhibit earlier and more durable resolution of their perforations. This can be visualized at the first postoperative endoscopy in which recanalization of small vessels overlying the temporalis fascia, as seen in Figure 3. The use of robust tissue with an overlying vascular plexus is analogous to anterolateral thigh fascia lata “rescue” free flaps used to restore vascularity to devascularized areas. The success of fascia lata free flaps is based upon maintenance of this vascular plexus, as shown in Figure 2B, and not the fascia itself, implying that revascularization through maintenance of these vascular channels in the temporalis fascia is likely key to success of this type of graft.^28^ Beyond free tissue transfer, the benefits of early vascular ingrowth have been demonstrated to promote successful healing of other grafts. Specifically, a study by Toriumi et al examining the survival of auricular composite grafts argued for the importance of maintaining perichondrium with its vascular channels and maximizing its contact with adjacent vascularized tissue as it enhances vascular ingrowth to the central components of the graft to promote successful graft survival.^29^ These results provide further evidence for the value of PAT in promoting the successful resolution of NSP. While our study was not specifically designed to compare the success of trilayer temporalis fascia interposition grafting with other NSP repair techniques, we do hypothesize that the careful harvesting of PAT associated with temporalis fascia does offer an advantage over other techniques.

As this study is retrospective in nature, it is not without limitations. The lack of standardized patient‐reported outcome measures with regard to nasal symptoms, such as the NOSE or sinonasal outcome test (SNOT‐22) limits the ability to draw objective conclusions. Evaluation of outcomes were thus assessed by physician examination and patient self‐reported symptoms. Finally, this study did not include a direct comparison with other commonly utilized NSP repair techniques, such as TPF grafting as this is not a technique utilized at our institution. Prospective studies assessing these techniques are necessary to fully conclude the superiority of one such technique over the other. Nevertheless, we believe that our results indicate a high degree of efficacy with limited morbidity.

## Conclusion

The use of a trilayer temporalis fascia interposition graft is a highly successful and reliable technique with low morbidity for the repair of NSPs. This technique is simple without the need for intranasal flaps, is associated with a high degree of perforation resolution and improvement in patient symptoms, and does not result in significant complications.

## Author Contributions

**Derek J. Vos**, data collection, data analysis, manuscript writing, manuscript editing; **Kristen A. Echanique**, manuscript writing, manuscript editing; **Amit Nag**, data collection, manuscript editing; **Stephen Hadford**, study design, data collection, manuscript editing; **Peter Ciolek**, study design, manuscript editing; **Dane J. Genther**, study conception, study design, manuscript editing.

## Disclosures

### Competing interests

None.

### Funding source

None.
